# Hormesis Effects of Nano- and Micro-sized Copper Oxide

**DOI:** 10.22037/ijpr.2019.13971.12030

**Published:** 2019

**Authors:** Majid Keshavarzi, Forouzan Khodaei, Asma Siavashpour, Arastoo Saeedi, Afshin Mohammadi-Bardbori

**Affiliations:** *Department of Pharmacology and Toxicology, School of Pharmacy, Shiraz University of Medical Sciences, Shiraz, Iran.*

**Keywords:** Copper oxide, Copper oxide nanoparticles, Hormesis effects, Mitohormesis, Mitotoxicity

## Abstract

The concerns about the possible risk of manufactured nanoparticles (NPs) have been raised recently. Nano- and micro-sized copper oxide (CO and CONP) are widely used in many industries. In this regard, *in-vitro* studies have demonstrated that CONP is a toxic compound in different cell lines. Despite their unique properties, NPs possess unexpected toxicity profiling relative to the bulk materials. This study was designed to examine and compare the toxic effects of CO and CONPs *in-vivo* and in isolated rat mitochondria. Male Wistar albino rats received 50 to 1000 mg/kg CO or CONP by gavage and several toxicological endpoints including biochemical indices and oxidative stress markers. Then, the pathological parameters in the multiple organs such as liver, brain, spleen, kidney, and intestine were assessed. Mitochondria were isolated from the rat liver and several mitochondrial indices were measured. The results of this study demonstrated that CO and CONP exhibited biphasic dose-response effects. CONPs showed higher toxicity compared with the bulk material. There were no significant changes in the results of CONP and CO in isolated rat liver mitochondria. The present studies provided more information regarding the hormetic effects of CO and CONPs *in-vivo* and in isolated rat mitochondria.

## Introduction

Metal oxides especially copper oxide nanoparticles (CONPs) have received growing attention due to the wide range of applications in recent years. Also, humans are constantly exposed to those particles occupationally or via consumer products. In order to lower the risk of exposure, it is necessary to have detailed accounts for proper risk assessment of chemical (1, 2).

Copper (CO) is one of the cofactors essential for various enzymes, cellular respiration, neurotransmitter regulation, collagen synthesis, and metabolism of nutrients (3). However, several studies have demonstrated that both forms of copper (CO and CONPs) provoke toxic responses at high doses in different biological systems including animals, various mammalian cell lines, plants, and bacteria (4-7).

Copper ions (CuCl_2_·2H_2_O) can cause severe pathological damages compared to CO in different organs of mice such as the kidney, liver, and spleen (8). Besides, high doses of CO via releasing CO ions is linked with a harmful effect on human health, living organisms, and environment (9). However, from *in-vitro* studies, it can be speculated that CONPs toxicity cannot be simply explained by the release of CO ion into the cell culture media (8, 10). CO and CONPs are able to interfere with the homeostasis of other metals and form reactive oxygen species that damage DNA and cellular components (11). CONPs have also been suggested to promote apoptosis via generation of reactive oxygen species (ROS) and activation of intracellular signaling through the mitochondrial-dependent pathway (12-16). 

Particle size has a direct relationship with many of the chemical features, including surface area, solubility, and reactivity that can strongly affect the toxicity of nanoparticles (11). Therefore, reducing the size leads to an increase in the surface area of nanoparticles that not only increases the accumulation of nanoparticles but also increases the reactivity and interaction with bio-molecules (17, 18). Similar to the other inorganic metals, CONPs also exhibits size and concentration-dependent toxicity (19). In this connection, when evaluating the toxicity of CONPs only a single physicochemical property such as size cannot be a final determination of predicted chemical toxicity; rather, the morphologies and the species-specific vulnerabilities of the cells should be taken in consideration, as well (20).

More than two centuries ago, hormesis became one of the most important issues in the field of pharmacology and toxicology and the biological response to the wide range of doses was characterized (21). In the field of toxicology, hundreds of substances illustrate biphasic dose responses, also called hormetic effects (22). Hormetic effects are common in a wide variety of inorganic minerals such as arsenic, cadmium, copper, lead, mercury, selenium, and zinc (23, 24). Besides, there are many examples of unusual dose-response for a large number of nanoparticles (25, 26).

To date, a large number of *in-vitro* studies have reported that CONPs at relatively high doses produce toxicity mainly based on the assessment of cell viability, ROS generation, and apoptosis. However, reports of *in-vivo* in different organs of animals and in isolated mitochondria are limited. This study was conducted to provide more evidence on the potential biphasic effects of CO and CONPs *in-vivo *and in isolated rat mitochondria. 

## Experimental

*Chemicals and physico-chemical characterization*

Bulk CO (CAS#: 1317-39-1) was purchased from Sigma-Aldrich, Germany. Spherical CONPs was purchased from US Research Nanomaterials, Inc. (CAS#: 1317-38-0). According to the manufacturer, the diameter of CONPs was 40 nm, purity was 99.9%, and the specific surface area was around 20 m^2^/g. 

The size distributions of CONPs (200 mg/mL) were measured after suspensions preparation using a laser diffraction particle size analyzer (Shimadzu, Model SALD-2101, Japan) at room temperature.

*Animals and treatments*

Male Wistar albino rats (180–220 g body weight) were obtained from the Center of Comparative and Experimental Medicine, Shiraz University of Medical Sciences. The animals were housed environmentally (t = 25 °C) and in an air humidity controlled room (60%). Then, they were kept on a standard laboratory diet and were maintained in a 12 h light-dark cycle for one week before the start of the experiments. They were treated according to the guideline of the Ethics Committee of Shiraz University of Medical Sciences. The animals were allowed to feed standard laboratory chow and tap water *ad libitum*.

The rats were divided into 11 groups of 8 animals randomly and received phosphate buffer saline (PBS) and different doses of CO and CONPs (50, 100, 250, 500, and 1000 mg/kg b.w) by gavage. They were treated for three consecutive days and at the end of the treatments were anesthetized by injecting 60 mg/kg thiopental. Next, the blood samples were collected for biochemical examinations. The brain, intestine, kidneys, spleen, and livers were quickly removed and homogenized for future examinations. 

*Preparation of mitochondria*

Rats liver was removed and mitochondria were isolated in a cold manitol solution containing 0.225 M D-manitol, 75 mM sucrose, and 0.2 mM ethylenediaminetetraacetic acid (EDTA), as described in (27). Approximately, 30 g of the minced liver was gently homogenized in a glass homogenizer with a Teflon pestle and then centrifuged at 700 × g for 19 min at 4 °C to remove nuclei, unbroken cells, and other non-sub-cellular tissues. The supernatant was centrifuged at 7000 × g for 20 min. The dark packed lower layer (heavy mitochondrial fraction) was re-suspended in the manitol solution and re-centrifuged twice at 7000 × g for 20 min. The heavy mitochondrial sediments were suspended in Tris solution containing 0.05 M Tris-HCl buffer (pH 7.4) 0.25 M sucrose, 20 mM KCl, 2.0 mM MgCl_2,_ and 1.0 mM Na_2_H PO_4_ at 4 °C before the assay.

*Analyses of mitochondrial viability*

In our study, the quantitative colorimetric method for determination of cell viability by MTT was modified for rat liver mitochondria suspension in tubes (succinate dehydrogenase activity) (27). 

*Analyses of Mitochondrial membrane potential (MMP)*

Rhodamine 123 was used to assess MMP (28, 29). The mitochondrial fractions were added into the reaction mixture containing 150 mM sucrose, 4 mM MgCl_2_, 5 mM potassium phosphate, and 30 mM KOH–HEPES (pH 7.4) in a total volume of 1 mL at 37 °C for 5 min. The reaction was initiated by adding 10 µL of 1 µM Rhodamine 123 and fluorescence was measured with excitation at 507 nm and emission at 527 nm (28). 

*Analyses of lipid peroxidation*

Levels of MDA were measured in different experimental groups. Briefly, the reaction mixture consists of 0.2 mL 8% SDS, 1.5 mL 20% trichloroacetic acid (TCA), and 0.6 mL of distilled water was prepared and mixed with 0.2 mL of tissue homogenate. The reaction was initiated by adding 1.5 mL of 1% thiobarbituric acid (TBA) and terminated by 10% trichloroacetic acid (TCA). The samples were centrifuged (3000 × g for 5 min) and the absorbance was measured at 532 nm (30).

*Analyses of GSH and GSSG contents*

Liver homogenates were mixed with 20% (w/v) trichloroacetic acid (TCA) and centrifuged at 10,000 × g for 20 min. The supernatant was removed and analyzed for reduced glutathione (31) by the 5,5’ dithiobis-2-nitrobenzoic acid (DTNB) recycling procedure (32). Total glutathione was determined in the supernatant after mixing with 1 mL of 5% sodium borohydride (NaBH_4_) and incubating at 45 °C for 60 min. The mixture was neutralized with 0.5 mL of 2.7 N HCl and total GSH was measured as described above. The absorbance at 412 nm was measured immediately after mixing. The GSH values were measured by extrapolation from a standard curve and GSSG expressed as GSH equivalents (33). GSH was normalized to cellular protein content. 

*Analyses of reactive oxygen species (ROS)*

ROS generation was evaluated using 2′,7′-dichlorofluorescein diacetate (DCFH-DA) dye as described previously (34). Briefly, 0.5 g of tissue was homogenized in a cold 40 mM Tris–HCl buffer (pH 7.4). For each sample, two parts containing 1 mL of 40 mM Tris–HCl buffer (pH 7.4) and 100 μL of the homogeneous mixture were prepared. In the first part, 10 µL of 1 µM DCFDA in methanol was added for ROS estimation and the same volume of methanol was added to the other part for control. The samples were incubated in a 37 °C for 30 min and fluorescence was read at excitation and emission wavelengths of 485 nm and 530 nm.

*Analyses of serum biochemical changes*

The animal blood samples were collected in the glass tube containing an anticoagulant substance (heparin). The biochemical parameters including blood urea nitrogen (BUN), creatinine (Cr), aspartate aminotransferase (SGOT), alanine aminotransferase (SGPT), total bilirubin (Bili Total), and lactate dehydrogenase (LDH) were measured by Biocon standard kits using automated Mannheim’s Erba XL 200 clinical chemistry analyzer.

*Protein concentration*

Sample protein concentrations were determined using the method developed by Bradford (35). Briefly, 100 µL of the suspensions were added to a 96-well plate and gently mixed with Bradford reagent. After 5 min, the absorbance was measured at 595 nm.

*Statistical analysis*

All values were expressed as mean ± SEM. To compare more than two experimental groups, One-way ANOVA followed by Tukey multiple comparison tests were used while to compare two experimental groups, two-tailed *t*-tests were used. *P*-values < 0.05 were considered statistically significant.

## Results

*Physico-chemical characterization of CONPs*

**Figure 1:**
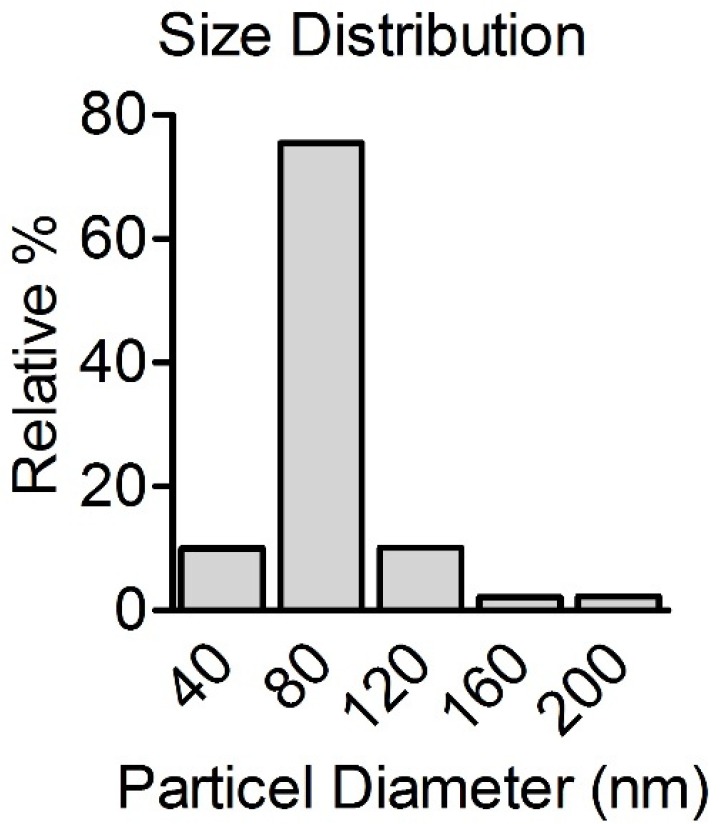
Size distribution of CONPs prepared in PBS

Figure 1 presents the size distribution of CONPs in suspension. When spherical CONPs nanopowder was suspended in the PBS, CONPs formed rapidly agglomerates. The results revealed that CONPs do not appear in the specified size according to the suppliers. It seems that in the suspension, CONPs tend to be aggregated. The mean size was 80 nm with more than 75% of particles.

*Effects of CO and CONPs on mitochondrial indices*

**Figure 2:**
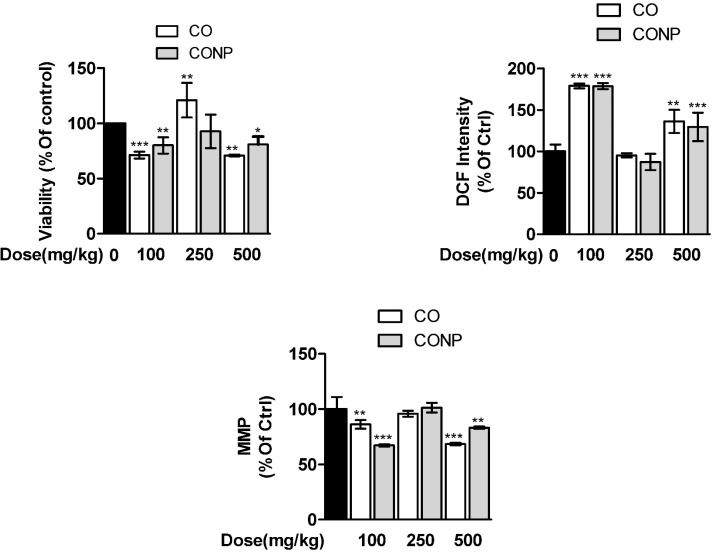
Effects of different doses of CO and CONPs on mitochondrial succinate dehydrogenase activity (SDA). Animals were exposed to PBS, 100, 250 and 500 mg/kg CO and CONPs for three consecutive days and at the end of the treatments, animals were anaesthetized and liver samples were collected for mitochondrial isolation. Mitochondria SDA were measured according to the materials and methods. Data are expressed as means ± SD. ^***^(*P* < 0.001) significantly different when compared with control alone. Effects of different doses of CO and CONPs on mitochondrial ROS formation. Animals were exposed to PBS, 100, 250 and 500 mg/kg CO and CONPs for three consecutive days and at the end of the treatments, animals were anaesthetized and liver samples were collected for mitochondrial isolation. ROS formation were measured according to the materials and methods. Data are expressed as means ± SD. ^***^(*P* < 0.001) significantly different when compared with control alone. Effects of different doses of CO and CONPs on mitochondrial membrane potential (MMP). Animals were exposed to PBS, 100, 250 and 500 mg/kg CO and CONPs for three consecutive days and at the end of the treatments, animals were anaesthetized and liver samples were collected for mitochondrial isolation. MMP were measured according to the materials and methods. Data are expressed as means ± SD. ^***^(*P* < 0.001) significantly different when compared with control alone

Quantitative data are presented in Figure 1. The mitochondrial succinate dehydrogenase activity decreased significantly in CO and CONPs 100 and 500 mg/kg treated groups compared with the control group. ROS formation was increased and MMP was declined by CO and CONPs 100 and 500 mg/kg approximately 78%-36%, and 13%-16%, respectively (Figure 1). There was no statistically significant difference between mito-toxicity profiling of nano- and micro-sized Copper oxide (Figure 2).

*Effects of CO and CONPs on oxidative stress biomarkers*

**Figure 3:**
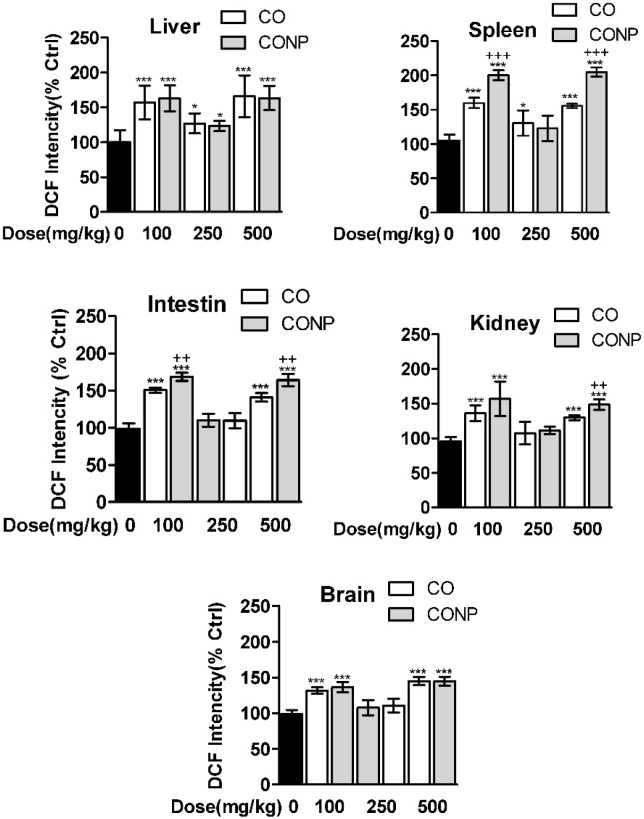
Effects of different doses of CO and CONPs rat organ ROS formation. Animals were exposed to PBS, 100, 250 and 500 mg/kg CO and CONPs for three consecutive days and at the end of the treatments, animals were anaesthetized and brain, intestine, kidney, spleen and livers were quickly removed and homogenized for ROS measurement. ROS formation were measured according to the materials and methods. Data are expressed as means ± SD. ^***^(*P* < 0.001) significantly different when compared with control alone

As shown in Figure 3, the ROS formation was significantly increased with all doses of CO and CONPs in liver, spleen, brain, and intestine compared with the control groups. Kidney ROS formation was seen in response to CO and CONPs 100 mg/kg and 500 mg/kg.

**Figure 4:**
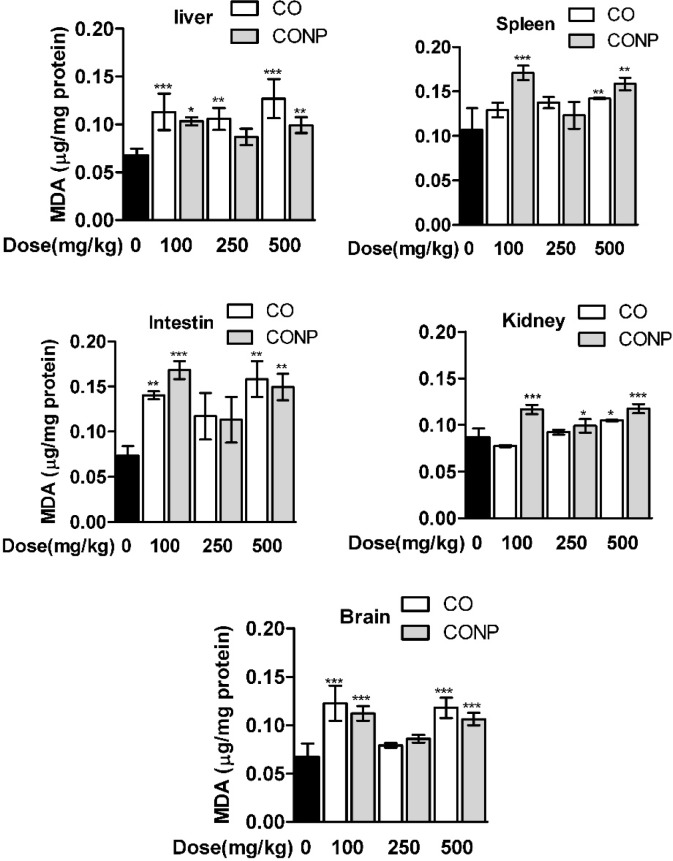
Effects of different doses of CO and CONPs rat organ MDA. Animals were exposed to PBS, 100, 250 and 500 mg/kg CO and CONPs for three consecutive days and at the end of the treatments, animals were anaesthetized and brain, intestine, kidney, spleen and livers were quickly removed and homogenized for MDA measurement. MDA were measured according to the materials and methods. Data are expressed as means ± SD. ^***^(*P* < 0.001) significantly different when compared with control alone

As shown in Table 1, the GSH contents were significantly decreased and GSSG was increased with the lowest and highest dose of CO and CONPs compared with the control groups in the brain, intestine, and spleen. However, in liver and kidney, GSH was declined and GSSG was increased with CO and CONPs 100 and 500 mg/kg while in kidney GSSG was only increased with CONPs 500 mg/kg. MDA content was significantly increased with the highest and lowest doses of CO and CONPs compared with the control groups (Figure 4).

*Effects of CO and CONPs on biochemical parameters*

Results of the enzyme activity analysis are shown in Table 2. 

Administration of all doses of CO and CONPs, except CO 250 mg/kg, to the rats caused a significant elevation in serum SGOT, SGPT, LDH, and Total Bili. Serum Cr at the highest and lowest doses of CONPs was increased compared to the control group. There was a significant difference between CO and CONPs treated groups (Table 2).

**Table 1:** Effects of different concentrations of CO and CONPs on reduced glutathione (31), total glutathione (Total GSH), oxidized glutathione (GSSG) and GSH/GSSG ratio. Animals were exposed to PBS, 100, 250 and 500 mg/kg CO and CONPs for three consecutive days and at the end of the treatments, animals were anaesthetized and brain, intestine, kidney, spleen and livers were quickly removed and homogenized for GSH and GSSG measurement. The GSH and GSSG were measured according to the materials and methods. Values are means ± SD for three independent experiments. a*P *< 0.05, b*P *< 0.01, c*P *< 0.001 compared to control

**GSH concentration (nmole/mg protein)**					
**Group Treatments (72 h)**		**GSH** **(nmole/mg protein)**	**Total GSH (nmole/mg protein)**	**GSSG** **(nmole/mg protein)**	**GSH/GSSG**
Brain	Control	105.08 ± 16.17	117.52 ± 19.78	12.44 ± 3.64	8.66
	CO100 (mg/kg)	78.21 ± 9.34a	136.08 ± 18.47	57.88 ± 15.53b	1.43
	CO250 (mg/kg)	87.23 ± 4.41	106.55 ± 9.96	19.33 ± 6.37	4.90
	CO500 (mg/kg)	82.61 ± 10.36	131.26 ± 10.96	48.64 ± 12.79b	1.80
	CONP100 (mg/kg)	72.25 ± 6.03b	122.84 ± 11.77	50.59 ± 13.51b	1.50
	CONP250 (mg/kg)	84.10 ± 7.70	99.43 ± 8.58	15.33 ± 1.20	5.49
	CONP500 (mg/kg)	79.27 ± 1.10a	128.58 ± 12.2	49.31 ± 3.2b	1.61
Intestine	Control	47.03 ± 6.88	69.44 ± 7.49	22.42 ± 1.80	2.10
	CO100 (mg/kg)	12.01 ± 4.23c	49.93 ± 2.89	37.92 ± 7.79b	0.34
	CO250 (mg/kg)	28.26 ± 6.01	58.12 ± 11.32	29.86 ± 5.54	1.16
	CO500 (mg/kg)	15.67 ± 2.82b	54.97 ± 3.55	39.31 ± 10.24a	0.48
	CONP100 (mg/kg)	24.08 ± 4.95a	55.11 ± 4.12	31.02 ± 2.48a	0.78
	CONP250 (mg/kg)	36.06 ± 9.04	60.44 ± 11.81	24.38 ± 2.96	1.46
	CONP500 (mg/kg)	5.69 ± 4.9c	51.05 ± 3.7	45.36 ± 2.4c	0.13
Kidney	Control	69.17 ± 9.03	72.48 ± 9.30	3.32 ± 0.27	20.80
	CO100 (mg/kg)	44.06 ± 1.88c	50.15 ± 0.79	6.10 ± 1.39	7.58
	CO250 (mg/kg)	60.61 ± 5.86	64.37 ± 6.58	3.76 ± 0.74	16.31
	CO500 (mg/kg)	43.75 ± 4.83c	48.98 ± 4.62	5.23 ± 0.80	8.53
	CONP100 (mg/kg)	46.36 ± 4.88c	51.79 ± 5.97	5.42 ± 1.46	8.89
	CONP250 (mg/kg)	60.79 ± 4.20	64.29 ± 4.45	3.50 ± 0.32	17.40
	CONP500 (mg/kg)	43.43 ± 2.2c	50.23 ± 3.5	6.80 ± 1.1b	6.38
Liver	Control	247.13 ± 30.94	253.90 ± 31.53	6.77 ± 0.67	36.48
	CO100 (mg/kg)	98.01 ± 14.71c	108.68 ± 15.89	10.67 ± 2.82	9.53
	CO250 (mg/kg)	193.13 ± 34.16	202.41 ± 34.49	9.27 ± 1.57	21.10
	CO500 (mg/kg)	180.51 ± 27.89a	196.34 ± 26.82	15.82 ± 1.21c	11.53
	CONP100 (mg/kg)	160.14 ± 10.73b	171.83 ± 11.87	11.69 ± 1.66a	13.83
	CONP250 (mg/kg)	226.82 ± 12.66	237.46 ± 15.34	10.64 ± 2.68	22.00
	CONP500 (mg/kg)	145.59 ± 10.5c	164.58 ± 13.3	19.00 ± 2.1c	7.66
Spleen	Control	51.39 ± 7.76	62.94 ± 3.57	11.55 ± 5.08	5.12
	CO100 (mg/kg)	13.26 ± 7.34c	41.87 ± 4.20	28.61 ± 4.04a	0.49
	CO250 (mg/kg)	35.47 ± 1.99	47.11 ± 4.08	11.64 ± 3.38	3.21
	CO500 (mg/kg)	16.69 ± 10.73c	44.44 ± 3.92	27.74 ± 8.69a	0.74
	CONP100 (mg/kg)	13.07 ± 3.65c	40.81 ± 7.86	27.74 ± 5.27a	0.47
	CONP250 (mg/kg)	37.76 ± 2.60	54.17 ± 5.90	16.41 ± 8.50	2.70
	CONP500 (mg/kg)	31.75 ± 2.5b	54.34 ± 6.5	22.59 ± 5.9a	1.41

**Table 2:** Effects of different concentrations of CO and CONPs on biochemical parameters. Animals were exposed to PBS, 100, 250 and 500 mg/kg CO and CONPs for three consecutive days and at the end of the treatments, animals were anaesthetized and blood samples were collected for biochemical examinations. Values are expressed as mean ± SD. a and c indicate significant differences (a*P *< 0.05; b*P *< 0.01; c*P *< 0.01) in exposed groups *vs. *control. BUN: blood urea nitrogen; Cr: creatinine; SGOT: aspartate aminotransferase; SGPT: alanine aminotransferase; Bili Total: total bilirubin; LDH: lactate dehydrogenase

**Groups Biochemical parameters**						
	**BUN (mg/dL)**	**Cr (mg/dL)**	**SGOT ( U/L)**	**SGPT (U/L)**	**Total Bili (mg/dL)**	**LDH (U/L)**
Control	28.3 ± 3.05	0.60 ± 0.11	115.33 ± 1.22	68.00 ± 7.21	0.20 ± 0 .001	129.66 ± 28.53
CO100 (mg/kg)	21.2 ± 3.40	0.62 ± 0.05	133.75 ± 2.70c	59.5 ± 1.28b	0.30 ± 0.001c	597.00 ± 5.69c
CO250 (mg/kg)	23.5 ± 5.97	0.57 ± 0.12	128.00 ± 1.07c	63.21 ± 1.27	0.23 ± 0.05	371.5 ± 28.11c
CO500 (mg/kg)	20.2 ± 4.11	0.65 ± 0.05	133.25 ± 2.42c	77.50 ± 1.27c	0.30 ± 0.001c	606.5 ± 14.75c
CONP100 (mg/kg)	21.2 ± 3.27	0.70 ± 0.01a	151.23 ± 2.03c	74.33 ± 1.85a	0.28 ± 0.008c	710.6 ± 8.44c
CONP250 (mg/kg)	25.0 ± 3.55	0.57 ± 0.05	126.00 ± 3.04c	76.00 ± 1.70b	0.24 ± 0.005a	425.75 ± 18.95c
CONP500 (mg/kg)	24.0 ± 4.24	0.75 ± 0.05a	133.25 ± 2.70c	76.00 ± 1.50b	0.25 ± 0.007b	700.75 ± 23.23c

## Discussion

In this study, the toxicity profiling of a micro and nano-sized copper oxide was evaluated in different organs of rat including liver, kidney, intestine, brain, and spleen using several oxidative stress biomarkers and biochemical parameters. In addition, mitotoxicity of CO and CONPs were evaluated in isolated mitochondria from the rat liver. We observed that CO and CONPs exhibited to some extent a U shape dose-response pattern (Figures 2-4 and Tables 1 and 2). 

In addition, their effects on isolated rat mitochondria, which include MTT assay, mitochondrial membrane potential, and ROS formation, were studied and similar mitohormesis effects were observed (Figure 2). Overall, *in-vivo* experiments showed a significant difference between toxicity profiling of CO and CONPs but mitotoxicity profiling of CONPs and CO was similar (Figures 2-4 and Tables 1 and 2). 

The mode of action of CO and CONPs has already been explained by chemical interaction with biomolecules and induction of oxidative stress (36, 37). Increasing ROS formation leads to the degradation of DNA, increased expression of the death receptors and cause mitochondrial dysfunction (37). In addition, as a very reactive redox chemical, CO in the presence of iron can give rise to hydroxyl radicals formation through Fenton reaction (38). 

In line with this result, studies have demonstrated that CONPs through the mitochondrial-dependent pathway in HepG2 cells can induce apoptosis cascade (39). In agreement with our data, caspase-3 apoptotic genes have shown to be up-regulated by exposure to CONPs along with reducing the mitochondrial membrane potential (39). From our mitochondrial experiments and the other relevant studies, it can be speculated that mitochondria are target organelle of CO and CONPs toxicity (Figure 2) (40). We suggest that CO and CONPs can interact directly with the mitochondria that are the main source of free radicals formation in the cells and cause oxidative damages (41). However, low intensity of ROS may lead to activation of signal transmission paths to initiate defense responses (42). Indeed, NPs can also impair the transfer of ions and electrons from the cell membrane and membrane of mitochondria (43-45).

It has been found that metals cause biphasic dose-response in cell culture and animal models (46-48). Our results are consistent with a previous finding on the U pattern dose response of NPs (49). The mechanisms that mediate hormesis effects are not well known. It has been suggested that the heat shock protein 70 (HSP70) family (50), as well as metallothionein (MT) proteins, are mediators of hormesis because the levels of those proteins are increased in response to heavy metals exposure (51, 52). Our results demonstrated that GSH/GSSG ratio is changed after CO and CONPs exposure. Glutathione is an important antioxidant enzyme that has a functional thiol group. In this connection, previous studies have shown that copper poisoning results from the reaction of metal with glutathione (53). Although some studies proposed the copper-catalyzed oxidation of glutathione (45), but the exact mechanism is unclear (54).

 The toxicity of the metal ions in mammalian systems is due to the chemical reaction of the ions with the cellular components such as structural proteins, enzymes, and membrane systems (55-57). 

The severity of toxicity is usually dependent on the metals accumulation in the target organs (55, 58). Comparison of bioavailability of copper ion and copper nanoparticles in a single oral dose (500 mg/kg) in the rats has indicated that both nanoparticles and copper ions in liver, kidney, and spleen can be accumulated (59). Heavy metals are able to accumulate in the vital organs such as the heart, brain, kidney, liver (60, 61), and bone (62, 63) and cause different clinical characteristics (64) such as growth retardation (55), various types of cancer (65, 66), kidney and liver damages (31), and impairment of immune system (67) and the other disorders (68-70). NPs also can cause harmful effects on the respiratory (71, 72), cardiovascular, and nervous systems (73).

In this study, we did not focus on the all aspect of CO and CONPs toxicity. It seems that CONPs cause more organ toxicity compared with bulk materials. Similar studies have demonstrated that NPs with the size smaller than 100 nm can easily enter the cell while those smaller than 40 and 30 nm can readily enter the nucleus and cross the blood-brain barrier, respectively (74). Small NPs have a tendency to form aggregation rather than single units, particularly under physiological conditions (75). NPs larger than 100 nm can be readily engulfed by the alveolar macrophages and those particles that are smaller than 100 nm tend to be aggregated and engulfed by the phagocytosis (76, 77). However, in non-phagocytes, size, shape, and the other physiochemical properties such as molecule surface charge can facilitate its internalization and affect NOPs induce organ toxicity (77-81).

## Conclusion

The present studies provided information regarding the hormetic effects of CO and CONPs *in-viv*o and in isolated rat mitochondria. We found that CONPs and CO have a similar pattern of toxicity. ROS formation was an early event leading to oxidative damage by CONPs and CO in different organs of the rats. The results of this study may provide more accurate information for a proper risk assessment of CONPs.
